# Paraoxonase 1 Activity and Renal Replacement Therapy for Chronic Renal Failure: A Meta-Analysis

**DOI:** 10.3390/jcm12155123

**Published:** 2023-08-04

**Authors:** Jun Watanabe, Kazuhiko Kotani, Yoshitaka Iwazu, Alejandro Gugliucci

**Affiliations:** 1 Division of Community and Family Medicine, Jichi Medical University, Tochigi 329-0498, Japan; m06105jw@jichi.ac.jp; 2 Division of Anti-Aging Medicine, Center for Molecular Medicine, Jichi Medical University, Tochigi 329-0498, Japan; iwazu@jichi.ac.jp; 3 Glycation, Oxidation and Disease Laboratory, Touro University California, Vallejo, CA 94592, USA

**Keywords:** arylesterase, chronic kidney disease, hemodialysis, paraoxonase, renal transplantation

## Abstract

Chronic renal failure (CRF) is associated with the development of cardiovascular disease (CVD). Paraoxonase 1 (PON1), an antioxidant enzyme, shows cardioprotective properties and has been proposed as a therapeutic marker for CRF. A systematic analysis of the literature assessing the association between PON1 activity and renal replacement therapy (RRT) of CRF is currently lacking. Therefore, we set out to perform a meta-analysis of the available data on PON1 in RRT of CRF. We searched three electronic databases for studies on PON1 activity in CRF patients with RRT such as hemodialysis (HD), peritoneal dialysis (PD), or renal transplantation (RTx), published before June 2023. A random-effects and network meta-analysis were performed. A total of 53 studies were eligibly identified. Compared to CRF patients without RRT, RTx patients had higher paraoxonase activity (standard mean difference (SMD), 1.76, 95% confidence interval (CI), 0.76–2.75), followed by HD (SMD, 0.73; 95% CI, 0.02–1.45) and PD patients. Likewise, RTx patients had higher arylesterase activity (SMD, 1.84, 95% CI, 0.18–3.50), followed by HD and PD patients. Also, paraoxonase activity was increased after HD (SMD, 0.59, 95% CI, 0.16–1.03). In conclusion, the overall data demonstrated that PON1 activity is higher in CRF patients with RRT, particularly RTx, followed by that of HD and PD. Measuring PON1 activity can also be included to the paraclinical toolbox for the management of RRT, in addition to the understanding of CRF-related pathophysiology. Regarding the selection of RRT types and their potential to prevent CVD, more research is required.

## 1. Introduction

With a high incidence and being a leading cause of mortality, cardiovascular disease (CVD) is a global health concern; accordingly, numerous risk factors have been studied in an effort to lower the risk [1,2]. Disorders of low-density lipoprotein (LDL) and high-density lipoprotein (HDL) are some of the risk factors for the development of CVD, specifically their concentrations in blood and qualitative dysfunction [2].

Paraoxonase 1 (PON1) is an esterase and lactonase enzyme which participates in antioxidant defenses and that is synthesized primarily by the liver and released into the blood circulation linked to HDL [3,4]. PON1 has paraoxonase and arylesterase activity because it hydrolyzes aromatic carboxylic acid esters like phenylacetate and organophosphate compounds like paraoxon. Paraoxon for paraoxonase is a “discriminating” substrate (with a polymorphic distribution of activity), whereas phenylacetate for arylesterase is a “nondiscriminating” substrate; thus, PON1 activity is reported in the literature as paraoxonase or arylesterase [3,4], while PON1′s lactonizing/lactonase activity is presumed to be the important one physiologically. Along with its antioxidant capabilities, PON1 has antiatherogenic effects that prevent the development of macrophage foam cells, including decreased cholesterol and oxidized lipid input as well as facilitation of macrophage cholesterol efflux [3,4]. We can see evidence supporting the protection of PON1 activity on CVD [3,4].

Chronic kidney disease/chronic renal failure (CRF) is a common pathological condition of renal damage, and renal replacement therapy (RRT) (i.e., renal replacement therapy including hemodialysis [HD], peritoneal dialysis [PD], renal transplantation [RTx]) is applied to the end-stage kidney disease [5]. Cardiovascular disease is a frequent occurrence in CRF [5], and remarkably, PON1 activity is inversely related to CVD in CRF patients even when they are receiving RRT, suggesting the important role of antioxidant status in the pathophysiology of CVD in the management of CRF [2,4,6]. The impact of therapeutic interventions, such as RRT, on antioxidant defenses should be more clarified.

Our previous meta-analysis revealed that CRF patients without RRT had lower PON1 activity (arylesterase and paraoxonase) compared to healthy individuals, indicating PON1 may serve as a biomarker in CRF in relation to CVD [7]. However, an analysis of the current literature on the effects of RRT on PON1 activity in CRF patients remains needed. Hence, the aim of this study was to conduct a systematic meta-analysis of the available clinical studies on the effects of all RRT types on PON1 activity in such patients.

## 2. Materials and Methods

This study was performed following standard protocols according to the Preferred Reporting Items for Systematic Review and Meta-analysis 2020 [8]. The study protocol was registered in PROSPERO (CRD42023389430).

### 2.1. Eligibility Criteria

The studies included in the analysis met the following criteria: they were comparative studies by cohort, case-control, and cross-sectional designs, and reported the data of PON1 activity in healthy subjects aged 18 years and older, CRF patients without RRT, and CRF patients with HD, PD, or RTx. CRF without RRT was defined as the estimated glomerular filtration rate, eGFR < 30 mL/min/1.73 m^2^ (according to the international guidelines) without any corresponding RRT [9].

### 2.2. Information Source and Search

To identify studies investigating PON1 activity in CRF patients with RRT, a two-step strategy was employed. Firstly, comprehensive searches were conducted in databases of MEDLINE, Embase, and the Cochrane Central Register of Controlled Trials. These were conducted until 1 June 2023 using the search engines of PubMed, Dialog, and Cochrane Library. The search terms included (“aryldialkylphosphatase” or “arylesterase” or “paraoxonase”) and (“renal insufficiency” or “chronic kidney failure” or “chronic renal insufficiency” or “kidney diseases” or “uremia” or “renal dialysis”) (Appendix A). There were no language restrictions applied during the search process.

### 2.3. Study Selection and Data Collection Process

In the screening process, independent reviewers (J.W. and K.K.) assessed the titles and abstracts of the identified studies. The eligibility assessment was then conducted based on the full texts of the selected studies, and the data were extracted. Any disagreements between the reviewers were resolved through discussions or by consulting an additional investigator (A.G.). The extracted data detailed the study design, study population, interventions, and outcomes. For the evaluation of risk of bias, independent reviewers (J.W. and K.K.) assessed the risk of bias using an 11-item checklist that was recommended by the Agency for Healthcare Research and Quality [10]. In case of any disagreements between the reviewers, they were discussed, and if necessary, a third reviewer (A.G.) acted as an arbiter.

### 2.4. Data Synthesis and Statistical Analysis

The network geometries are visually illustrated with circles representing the surgical procedure as nodes in the network, lines depicting direct comparisons using studies, and the width of the lines indicating the number of studies included in each comparison, which is also represented by corresponding numbers.

The standard mean differences (SMDs) and corresponding 95% confidence intervals (CIs) for paraoxonase and arylesterase activity, both continuous variables, were pooled in our analysis based on the Cochrane handbook [11]. Group-level data were utilized, and a random-effects Frequentist network meta-analysis model was employed to synthesize the effect sizes from the included studies. The normal likelihood was applied for continuous outcomes, and a normal prior was used. To account for correlations induced by multi-group studies, multivariable distributions were utilized. The variance in the random-effects distribution, referred to as the heterogeneity variance, was considered to capture the effects of both cross-study and within-comparison variability on treatment effects. The MetaInsight software version 4.2.0 was used for the analysis [12]. We also calculated the SMDs for paraoxonase activity before and after HD. The threshold of significance was *p* < 0.05.

## 3. Results

**Figure 1:**
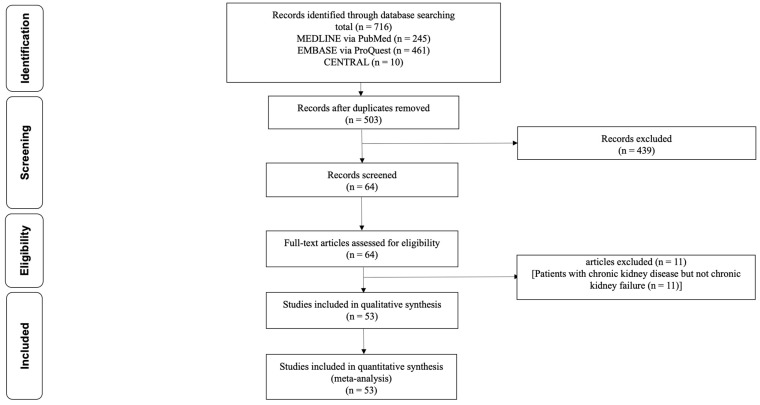
Flow diagram of selection of articles that reported the PON1 in patients with chronic renal failure who were treated with hemodialysis, peritoneal dialysis, or renal transplantation. PON1, paraoxonase 1.

Figure 1 illustrates the process of study selection.

Following the removal of duplicate records, a total of 503 unique records were identified. After the initial screening process, 439 records were excluded based on predetermined criteria, resulting in 64 remaining records. During the secondary screening, 11 studies involving patients with CKD but not CRF (as eGFR ≥ 30 mL/min/1.73 m^2^) were excluded. Ultimately, a total of 53 studies were included in the analysis for further evaluation and synthesis [13,14,15,16,17,18,19,20,21,22,23,24,25,26,27,28,29,30,31,32,33,34,35,36,37,38,39,40,41,42,43,44,45,46,47,48,49,50,51,52,53,54,55,56,57,58,59,60,61,62,63,64,65]. Of 53 studies, 47 studies reported PON1 activity in CRF patients with and without RRT. Two studies reported paraoxonase activity before and after HD, while four studies reported PON1 activity in CRF patients with and without RRT as well as paraoxonase activity before and after HD.

Table 1 shows a summary of the study characteristics in CRF patients with RRT. A total of 48 studies involving 7898 patients (53 years of mean age) reported paraoxonase activity, while 22 studies involving 3942 patients (49 years of mean age) reported arylesterase activity. The overall risk of bias was median 7, range 3–8, using an 11-item checklist by AHRQ in Appendix B.

**Figure 2:**
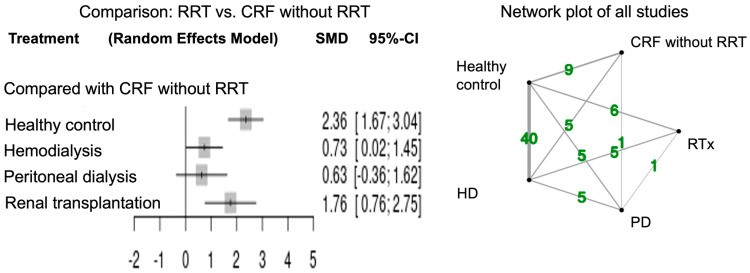
Forest and network plots of paraoxonase activity in all studies compared with CRF without RRT. Green number indicates the study numbers. CRF, chronic renal failure; RRT, renal replacement therapy.

Figure 2 shows the forest plots for network meta-analysis and the network plot of all studies that measured paraoxonase activity. The network meta-analysis of paraoxonase activity was performed in 48 studies. Healthy controls had higher activity relative to CRF patients in accordance with the previous report [7]. Paraoxonase activity was significantly higher in CRF patients with RTx (SMD 1.76; 95% CI, 0.76–2.75) and HD (SMD 0.73; 95% CI, 0.02–1.45) compared to those without RRT. Paraoxonase activity tended to be high in CRF patients with PD compared to those without RRT (SMD 0.63; 95% CI, −0.36–1.62).

**Figure 3:**
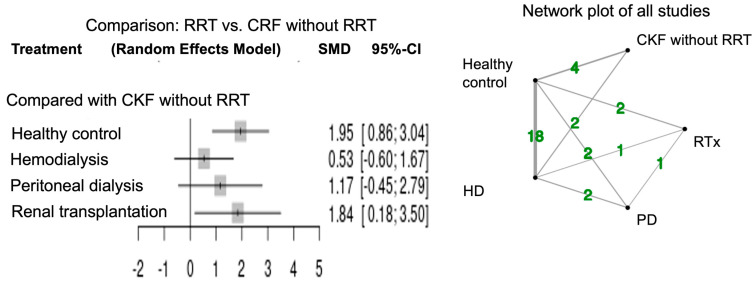
Forest plot and network plots of arylesterase activity in all studies compared with CRF without RRT. Green number indicates the study numbers. CRF, chronic renal failure; RRT, renal replacement therapy.

Figure 3 shows the forest plots for network meta-analysis and the network plot of all studies measuring arylesterase activity. The network meta-analysis of arylesterase activity was performed in 22 studies. Healthy controls had higher activity relative to CRF patients in agreement with the previous report [7]. Arylesterase activity was significantly higher in CRF patients with RTx (SMD 1.84; 95% CI, 0.18–3.50) compared to those without RRT. Arylesterase activity tended to be high in CRF patients with PD (SMD 1.17; 95% CI, −0.45–2.79) and HD (SMD 0.53; 95% CI, −0.60–1.67) compared to those without RRT.

**Figure 4:**
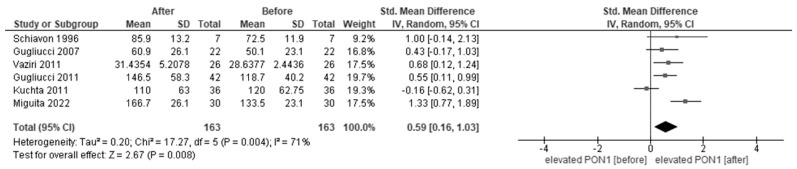
Forest plot of paraoxonase activity before and after hemodialysis [29,33,34,51,65].

Figure 4 shows the forest plots of paraoxonase activity in CRF patients before and after HD. In the meta-analysis, six studies reported paraoxonase activity in patients before and after HD. Paraoxonase activity was increased after HD (SMD, 0.59, 95% CI, 0.16–1.03) compared to that before HD.

## 4. Discussion

Given the importance of PON1 for CVD in CRF patients with RRT, the present comprehensive meta-analysis of studies was carried out to assess the effect of various RRT types on PON1 activity [2,4,6]. The analysis corroborated the earlier finding that healthy controls have higher PON1 activities than CFK patients [7]. Importantly, we observed that paraoxonase and arylesterase activities were highest in CRF patients receiving RTx, followed by higher activity levels in patients under HD and PD as compared to those not receiving RRT. Additionally, paraoxonase activity was increased in CRF patients during HD when measured before and after this treatment modality. This might provide valuable insights into the use of PON1 activity in assessing the CRF-related pathophysiology as a surrogate marker of CVD and the potential guidance of therapeutic strategies for CRF patients.

**Figure 5:**
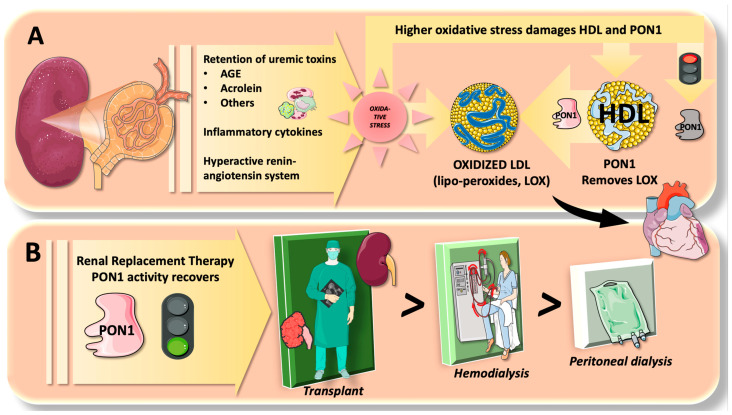
Paraoxonase 1 dysfunction in chronic renal failure (CRF) and the effects of renal replacement therapy. (**A**) Deficient filtration in CRF leads to retention of uremic toxins such as advanced glycation endproducts (AGE), acrolein, and others, together with inflammatory cytokines which produce a burden of excessive oxidative stress. Lipoperoxides (LOX) are formed in low-density lipoprotein (LDL), which renders LDL more atherogenic. These are eliminated in part by paraoxonase 1 (PON1) in high-density lipoprotein (HDL). Excess oxidative stress, however, can impair PON1 activity; thus, PON1 activity is consistently lower in CRF. (**B**). The effect of renal replacement therapy (RRT) as shown in the present meta-analysis is positive to restore PON1 activity; namely, renal transplantation (RTx) exhibits the highest effect, followed by hemodialysis (HD) and peritoneal dialysis (PD). The figure was partly generated using Servier Medical Art, provided by Servier, licensed under a Creative Commons Attribution 3.0 unported license.

As we summarize in Figure 5, “uremic” toxins, inflammatory cytokines, and hypertension with an active renin-angiotensin system cause excessive oxidative stress and decreased antioxidant defenses, which are generally recognized factors worsening CRF prognosis [66]. RRT can improve the situation by eliminating toxic substrates and alleviating a stressed-out system. The present meta-analysis results suggest that this may help to partially explain why PON1 activity was recovered. As seen in the present study, the degree of PON1 recovery differed depending on RRT types. Different mechanisms may be thus at work. For instance, the HD procedure by the dialysis machine [67] employs heparin to avoid clotting by contact with tubing and membranes. When heparin is employed, lipoprotein lipase is activated, which enhances very-low-density lipoprotein (VLDL) catabolism and an increase in HDL particles [68,69]. Along with the aggravating factors being removed, this may also contribute to an increase in PON1. On the other hand, the HD technique involves some aspects of the oxidative burden due to bioincompatible dialyzer, iron infusion, existence of anemia, and loss of antioxidants [70,71,72].

Next, alternative to HD, PD utilizing the patient’s own peritoneum as a filter is a less invasive as a comparatively biocompatible type of RRT, which may show a reduction in oxidative stress mainly due to low pH, high lactate content, high osmolarity, high glucose concentration, and related degradation products [73]. However, because of the high glucose load and the consequent metabolic derangements associated with PD, there is still an elevated risk of CVD with the oxidative burden by this modality [74,75]. Being an HDL-associated molecule, PON1 can be affected by dysfunctional triglyceride-rich lipoprotein catabolism enhanced by the insulin resistance induced by the glucose load [3,4].

Finally, RTx shows the best results in PON1 activity recovery in the present meta-analysis. This appeared to be consistent with the lowest mortality rates with a high quality of life achieved by this modality compared to HD and PD [76]. Although there are not very many studies comparing the responses to oxidative stress and antioxidant defense across RRT types, one study comparing PD and HD found that PD provided better antioxidant protection than HD when measuring superoxide dismutase, catalase, glutathione peroxidase, glutathione reductase, and glucose-6-phosphate dehydrogenase [77]. According to further research on RRT types, RTx removes oxidative stress-induced chemicals more effectively than HD and PD when measuring advanced glycation end products such as uremic toxins [78]. Despite a dearth of research evaluating PON1 activity across different RRT types, the results of our present meta-analysis, which found that CRF patients with RTx had the highest PON1 activity followed by those on HD and PD, are seemingly in agreement with some of those of earlier studies [77,78].

Some limitations apply to the present investigation. First, the methods of RRT (such as the devices and modes of HD) in the studies included on the meta-analysis might be heterogeneous since there were not unified study protocols. For instance, it remains to be a matter of consideration whether low- and high-flux membrane permeability of HD affects PON1 activity. Furthermore, the methods applicable to RRT are under continuous improvement. Second, the association of patient profiles related to RRT with PON1 activity was not fully described in the respective studies for the meta-analysis. For instance, time after the dialysis or transplantation can be informative as PON1 activity was reported to decrease with an increase in dialysis vintage (time on dialysis in years) [56,79]. Third, the association of lifestyle factors (e.g., diets) [80,81] and prescribed drugs [82] with PON1 activity was not explored in the included studies. In this case, therapies using antioxidants (e.g., vitamins B, C, D, and E, coenzyme Q10, L-carnitine, a-lipoic acid, curcumin, green tea, flavonoids, polyphenols, omega-3 polyunsaturated fatty acids, trace elements, N-acetylcysteine) might also be considered as the antioxidant supplementation is being studied to ameliorate oxidative stress in patients receiving HD and PD [83]. Forth, the effect of PON1 activity with RRT on cardiovascular events was not examined in the available studies. Finally, data on lactonase activity of PON1 was not included since the available data were so minimal. In future research, lactonase activity might be necessary to be included. Measurement assays of PON1 activity are not yet standardized (see units and measured values as shown in Table 1). Besides the need for their standardization, measuring PON1 activity in CRF patients with RRT may pave the way for more research along three axes: mechanistic studies, prospective cohort studies for outcomes, and intervention studies to boost PON1 function.

## 5. Conclusions

According to the overall findings, this meta-analysis provides robust information to attest that CRF patients with RRT, especially RTx, could have higher PON1 activity, followed by HD and PD. Measuring PON1 activity can be also included to the paraclinical toolbox for the management of RRT, in addition to the understanding of CRF-related pathophysiology. Further studies are thus warranted in relation to the choice of RRT types and the protective effect on CVD in this population.

## Figures and Tables

**Table 1:** Summary of the articles on PON1 activity included.

Authors [Ref No.]	Year	Country	Subject No.	Age	Unit	Healthy Controls	CRF without RRT	CRF with Hemodialysis	CRF with Peritoneal Dialysis	CRF with Renal Transplantation
**Paraoxonase**										
Schiavon [65]	1996	Italy	264	56	U/L	146.3 ± 51.0	–	93.0 ± 32.2	–	–
Hasselwander [64]	1998	Northern Ireland	78	54	U/L	139.5 ± 36.1	–	95.7 ± 26.8	–	–
Paragh [63]	1999	Hungary	342	48	U/mL	188.1 ± 59.0	–	101.4 ± 30.1	–	161.5 ± 35.4
Itahara [62]	2000	Japan	232	65	μmol/min/L	155.0 ± 57.0	–	97.0 ± 43.0	–	–
Juretić [61]	2001	Croatia	214	39	U/L	251.0 ± 143.0	–	167.0 ± 105.0	–	–
Ak [58]	2002	Turkey	55	44	nmol/min/mL	30.9 ± 19.4	–	14.4 ± 11.0	–	–
Schiavon [59]	2002	Italy	166	61	U/L	110.0 ± 160.0	–	73.5 ± 91.0	–	–
Suehiro [60]	2002	Japan	184	64	μmol/min/L	153.4 ± 54.7	–	94.7 ± 43.3	–	–
Dirican [56]	2004	Turkey	98	47	U/L	178.0 ± 79.0	140.0 ± 65.0	128.0 ± 51.0	–	–
Sutherland [57]	2004	New Zealand	19	48	μmol/mL/min	88.0 ± 16.0	–	56.0 ± 16.0	–	–
Kalogerakis [55]	2005	New Zealand	45	61	U/mL	125.2 ± 24.0	–	87.8 ± 25.7	–	–
Jurek [53]	2006	Poland	60	60	U/L	73.5 ± 43.3	–	42.7 ± 27.9	–	–
Krishnaswamy [54]	2006	Greece	75	35–65	U/L	137.0 ± 18.7	–	88.0 ± 14.4	130.0 ± 16.5	–
Dirican [50]	2007	Turkey	126	47	U/L	200.1 ± 98.3	–	124.7 ± 51.4	–	–
Gugliucci [51]	2007	USA	52	62	U/L	86.2 ± 25.2	–	50.1 ± 23.1	–	–
Horoz [52]	2007	Turkey	72	48	U/L	258.7 ± 38.8	–	146.8 ± 30.1	–	–
Atamer [42]	2008	Turkey	60	53	U/L	376.2 ± 72.4	231.1 ± 34.0	–	–	–
Dronca [43]	2008	Romania	50	51	U/L	572.6 ± 78.7	258.3 ± 42.5	–	–	–
Ferretti [44]	2008	Italy	92	66	U/mL	2316.9 ± 225.3	–	476.3 ± 52.4	–	–
Göçmen [45]	2008	Turkey	85	42	U/L	486.5 ± 24.6	–	–	392.7 ± 21.4	–
Lahrach [46]	2008	Morocco	209	45	U/mL	138.3 ± 82.5	–	117.0 ± 84.6	–	–
Prakash [47]	2008	India	130	53	U/L	192.5 ± 31.3	88.7 ± 38.9	60.0 ± 36.5	–	–
Saeed [48]	2008	Egypt	90	42	U/L	180.6 ± 27.7	141.7 ± 22.4	133.2 ± 19.2	–	–
Senol [49]	2008	Turkey	56	41	U/L	215.0 ± 108.0	–	113.0 ± 51.0	–	–
Moradi [39]	2009	USA	45	51	Ku/L	108.4 ± 11.7	–	94.8 ± 33.0	–	–
Paragh [40]	2009	Hungary	1326	48	U/L	111.1 ± 6.2	–	–	–	124.0 ± 14.3
Varga [41]	2009	Hungary	355	47	U/L	188.0 ± 62.0	–	64.6 ± 16.2	–	110.1 ± 23.6
Abdin [35]	2010	Egypt	50	54	U/mL	39.7 ± 12.4	14.3 ± 6.1	–	–	–
Moradi [37]	2010	USA	32	53	Ku/L	150.4 ± 44.7	–	95.7 ± 38.6	–	–
Rajković [38]	2010	Croatia	158	56	U/L	274.0 ± 148.5	–	159.0 ± 97.8	–	–
Emre [28]	2011	Turkey	54	38	U/mL	–	–	–	62.1 ± 41.6	107.6 ± 82.7
Gugliucci [29]	2011	Japan	91	63	U/L	357.0 ± 189.5	–	87.1 ± 20.5	–	–
Horoz [30]	2011	Turkey	71	49	U/L	245.0 ± 64.5	–	78.0 ± 63.0	–	–
Johnson-Davis [31]	2011	USA	47	18–84	U/L	281.3 ± 154.5	–	114.0 ± 58.4	192.9 ± 130.6	–
Kimak [32]	2011	Poland	193	46	U/L	142.0 ± 122.0	–	78.0 ± 63.5	–	115.0 ± 109.8
Kuchta [33]	2011	Poland	157	58	U/L	170.0 ± 95.8	113.5 ± 48.4	120.0 ± 62.8	100.0 ± 65.5	–
Cacciagiú [23]	2012	Argentina	85	50	U/mL	357.0 ± 189.5	–	276.0 ± 211.5	–	–
Gbandjaba [25]	2012	Morocco	175	60	U/mL	68.9 ± 99.5	–	39.7 ± 33.9	–	–
Ribeiro [27]	2012	Portugal	205	66	nmol of p-nitrophenol/mL/min	466.8 ± 53.0	–	362.6 ± 51.8	–	–
Sztanek [24]	2012	USA	249	52	U/L	99.4 ± 46.6	–	46.8 ± 17.4	–	70.2 ± 27.6
Kolarz [21]	2013	Poland	155	54	U/L	–	–	144.9 ± 34.2	79.0 ± 7.0	–
Locsey [22]	2013	Hungary	258	60	U/L	137.0 ± 82.1	–	58.0 ± 36.7	–	81.0 ± 46.5
Abdallah [19]	2017	Egypt	96	57	U/L	164.3 ± 61.5	–	82.1 ± 31.6	–	–
Coimbra [16]	2019	Portugal	216	71	nmol of p-nitrophenol/mL/min	413.0 ± 30.0	–	405.0 ± 41.5	–	–
Suematsu [18]	2019	USA	523	55	kU/L	135.2 ± 77.2	–	77.2 ± 35.8	–	–
Sridevi [17]	2021	India	123	20–60	U/L	383.0 ± 41.5	160.1 ± 9.4	188.4 ± 65.8	–	–
Jose [13]	2022	India	152	51	U/L	360.0 ± 24.7	146.2 ± 80.6	–	–	–
Szentimrei [15]	2022	Hungary	147	49	U/L	83.0 ± 30.0	–	46.0 ± 33.3	55.7 ± 32.0	–
**Arylesterase**										
Hasselwander [64]	1998	Northern Ireland	78	54	U/L	64.3 ± 17.0	–	50.4 ± 14.9	–	–
Paragh [63]	1999	Hungary	342	48	U/mL	125.0 ± 8.8	–	75.0 ± 14.4	95.0 ± 16.3	–
Itahara [62]	2000	Japan	232	65	mmol/min/L	92.0 ± 22.0	–	71.0 ± 20.0	–	–
Juretić [61]	2001	Croatia	214	39	kU/L	106.0 ± 38.0	–	52.0 ± 18.0	–	–
Suehiro [60]	2002	Japan	184	64	mmol/min/L	91.4 ± 22.0	–	71.8 ± 20.4	–	–
Dirican [56]	2004	Turkey	98	47	kU/L	84.0 ± 28.0	66.0 ± 19.0	63.0 ± 21.0	–	–
Jurek [53]	2006	Poland	58	60	U/mL	175.5 ± 35.3	–	70.4 ± 15.3	–	–
Dirican [56]	2007	Turkey	126	47	kU/L	74.1 ± 16.4	86.4 ± 31.3	–	–	–
Horoz [52]	2007	Turkey	72	49	kU/L	148.9 ± 22.8	–	90.5 ± 45.3	–	–
Dronca [43]	2008	Romania	50	51	U/L	93.4 ± 5.5	58.4 ± 1.9	–	–	–
Saeed [48]	2008	Egypt	90	42	kU/L	103.7 ± 14.8	78.2 ± 18.2	77.3 ± 12.7	–	–
Senol [49]	2008	Turkey	56	41	kU/L	75.0 ± 34.0	–	48.0 ± 15.0	–	–
Paragh [40]	2009	Hungary	1326	47	U/mL	85.5 ± 2.5	–	–	–	87.1 ± 5.3
Kannampuzha [36]	2010	Canada	30	41	U/mL	189.2 ± 20.4	–	128.0 ± 18.8	–	–
Emre [28]	2011	Turkey	54	38	U/mL	–	–	–	27.1 ± 23.3	45.8 ± 19.7
Gugliucci [29]	2011	Japan	91	63	U/L	176.6 ± 22.3	–	146.5 ± 30.8	–	–
Horoz [30]	2011	Turkey	71	49	U/L	237.0 ± 15.8	–	134.0 ± 39.0	–	–
Mahrooz [26]	2012	Iran	49	56	μU/mL	46.8 ± 26.9	–	50.0 ± 18.0	–	–
Locsey [22]	2013	Hungary	258	60	U/L	90.4 ± 20.7	–	103.7 ± 12.8	–	96.3 ± 16.2
Gugliucci [20]	2014	Japan	84	63	U/L	175.2 ± 32.2	–	146.2 ± 31.1	–	–
Szentimrei [15]	2022	Hungary	214	49	U/L	133.3 ± 29.7	–	111.8 ± 24.5	132.6 ± 30.1	–

CRF, chronic renal failure; RRT, renal replacement therapy.

## Data Availability

All data relevant to the study are included in the article.

## Appendix A

MEDLINE via PubMed
#1. “Aryldialkylphosphatase”[Mesh]
#2. “aryldialkylphosphatase”[tiab]
#3. “arylesterase”[tiab]
#4. “paraoxonase”[tiab]
#5. #1 OR #2 OR #3 OR #4
#6. “Renal Insufficiency”[Mesh]
#7. “Kidney Failure, Chronic”[Mesh]
#8. “Renal Insufficiency, Chronic”[Mesh]
#9. “Kidney Diseases”[Mesh]
#10. “Uremia”[Mesh]
#11. “end-stage renal”[tiab] OR “end-stage kidney”[tiab] OR “endstage renal”[tiab] OR “endstage kidney”[tiab]
#12. “ESRF”[tiab] OR “ESKF”[tiab] OR “ESRD”[tiab] OR “ESKD”[tiab]
#13. “chronic kidney”[tiab] OR “chronic renal”[tiab]
#14. “CKF”[tiab] OR “CKD”[tiab] OR “CRF”[tiab] OR “CRD”[tiab]
#15. “predialysis”[tiab] OR “predialysis”[tiab]
#16. “Renal Dialysis”[Mesh]
#17. ”hemodialysis”[tiab] OR “haemodialysis”[tiab]
#18. ”hemofiltration”[tiab] OR “haemofiltration”[tiab]
#19. ”hemodiafiltration”[tiab] OR “hemodiafiltration”[tiab]
#20. ”dialysis”[tiab] OR “dialysis”[tiab]
#21. “CAPD”[tiab] OR “CCPD”[tiab] OR “APD”[tiab]
#22. #6 OR #7 OR #8 OR #9 OR #10 OR #11 OR #12 OR #13 OR #14 OR #15 OR #16 OR #17 OR #18 OR #19 OR #20 OR #21
#17. #5 AND #22

Embase via Proquest
S1 EMB.EXACT.EXPLODE(“aryldialkylphosphatase”)
S2 ab(aryldialkylphosphatase) OR ti(aryldialkylphosphatase)
S3 ab(arylesterase) OR ti(arylesterase)
S4 ab(paraoxonase) OR ti(paraoxonase)
S5 S1 OR S2 OR S3 OR S4
S6. EMB.EXACT.EXPLODE(“kidney failure”)
S7. EMB.EXACT.EXPLODE(“chronic kidney failure”)
S8. EMB.EXACT.EXPLODE(“chronic kidney failure”)
S9. EMB.EXACT.EXPLODE(“kidney disease”)
S10. EMB.EXACT.EXPLODE(“uremia”)
S11. ab(end-stage renal) OR ti(end-stage renal) OR ab(end-stage kidney) OR ti(end-stage kidney) OR ab(endstage renal) OR ti(endstage renal) OR ab(endstage kidney) OR ti(endstage kidney)
S12. ab(ESRF) OR ti(ESRF) OR ab(ESKF) OR ti(ESKF) OR ab(ESRD) OR ti(ESRD) OR ab(ESKD) OR ti(ESKD)
S13. ab(chronic kidney) OR ti(chronic kidney) OR ab(chronic renal) OR ti(chronic renal)
S14. ab(CKF) OR ti(CKF) OR ab(CKD) OR ti(CKD) OR ab(CRF) OR ti(CRF) OR ab(CRD) OR ti(CRD)
S15. ab(predialysis) OR ti(predialysis) OR ab(predialysis) OR ti(predialysis)
S16. EMB.EXACT.EXPLODE(“hemodialysis”)
S17. ab(hemodialysis) OR ti(hemodialysis) OR ab(haemodialysis) OR ti(haemodialysis)
S18. ab(hemofiltration) OR ti(hemofiltration) OR ab(haemofiltration) OR ti(haemofiltration)
S19. ab(hemodiafiltration) OR ti(hemodiafiltration) OR ab(hemodiafiltration) OR ti(hemodiafiltration)
S20. ab(dialysis) OR ti(dialysis) OR ab(dialysis) OR ti(dialysis)
S21. ab(CAPD) OR ti(CAPD) OR ab(CCPD) OR ti(CCPD) OR ab(APD) OR ti(APD)
S22. S6 OR S7 OR S8 OR S9 OR S10 OR S11 OR S12 OR S13 OR S14 OR S15 OR S16 OR S17 OR S18 OR S19 OR S20 OR S21
S23. S5 AND S22

CENTRAL via Cochrane Library
#1. MeSH descriptor: [Aryldialkylphosphatase] explode all trees
#2. aryldialkylphosphatase:ti,ab
#3. arylesterase:ti,ab
#4. paraoxonase:ti,ab
#5. #1 OR #2 OR #3 OR #4
#6. MeSH descriptor: [Renal Insufficiency] explode all trees
#7. MeSH descriptor: [Kidney Failure, Chronic] explode all trees
#8. MeSH descriptor: [Renal Insufficiency, Chronic] explode all trees
#9. MeSH descriptor: [Kidney Diseases] explode all trees
#10. MeSH descriptor: [Uremia] explode all trees
#11. “end-stage renal”:ti,ab OR “end-stage kidney”:ti,ab OR “endstage renal”:ti,ab OR “endstage kidney”:ti,ab
#12. ESRF:ti,ab OR ESKF:ti,ab OR ESRD:ti,ab OR ESKD:ti,ab
#13. “chronic kidney”:ti,ab OR “chronic renal”:ti,ab
#14. CKF:ti,ab OR CKD:ti,ab OR CRF:ti,ab OR CRD:ti,ab
#15. predialysis:ti,ab OR predialysis:ti,ab
#16. MeSH descriptor: [Renal Dialysis] explode all trees
#17. hemodialysis:ti,ab OR haemodialysis:ti,ab
#18. hemofiltration:ti,ab OR haemofiltration:ti,ab
#19. hemodiafiltration:ti,ab OR hemodiafiltration:ti,ab
#20. dialysis:ti,ab OR dialysis:ti,ab
#21. “CAPD”[tiab] OR “CCPD”[tiab] OR “APD”[tiab]
#22. #6 OR #7 OR #8 OR #9 OR #10 OR #11 OR #12 OR #13 OR #14 OR #15 OR #16 OR #17 OR #18 OR #19 OR #20 OR #21
#17. #5 AND #22

## Appendix B

**Table A1:** Details of Risk of Bias Assessment.

Authors [Reference No.]	Define the Source of Information	List Inclusion and Exclusion Criteria for Exposed and Unexposed Subjects	Indicate Time Period Used for Identifying Patients	Indicate Whether or Not Subjects Were Consecutive If Not Population-Based	Indicate if Evaluators of Subjective Components of Study Were Masked	Describe Any Assessments Undertaken for Quality Assurance Purposes	Explain Any Patient Exclusions from Analysis	Describe Any Assessments Undertaken for Quality Assurance Purpose	If Applicable, Explain How Missing Data Were Handled in the Analysis	Describe How Confounding Was Assessed and/or Controlled	Summarize Patient Response Rates and Completeness of Data Collection	Score
Schiavon [65]	0	0	0	0	1	1	1	1	-	1	-	5
Hasselwander [64]	1	0	0	0	1	1	1	1	-	1	-	6
Paragh [63]	0	0	0	0	1	1	1	1	-	1	-	5
Itahara [62]	1	1	1	0	1	1	1	1	-	1	-	8
Juretić [61]	1	1	0	0	1	1	1	1	-	1	-	7
Ak [58]	1	1	0	0	1	1	1	1	-	1	-	7
Schiavon [59]	0	0	0	0	1	1	1	1	-	1	-	5
Suehiro [60]	0	0	0	0	1	1	1	1	-	1	-	5
Dirican [56]	1	1	0	0	1	1	1	1	-	1	-	7
Sutherland [57]	1	1	0	0	1	1	1	1	-	1	-	7
Kalogerakis [55]	1	1	0	0	1	1	1	1	-	1	-	7
Jurek [53]	0	1	1	0	1	1	1	1	-	1	-	7
Krishnaswamy [54]	1	0	0	0	1	1	1	1	-	1	-	6
Dirican [50]	1	1	0	0	1	1	1	1	-	1	-	7
Gugliucci [51]	1	1	0	0	1	1	1	1	-	1	-	7
Horoz [52]	1	1	0	0	1	1	1	1	-	1	-	7
Atamer [42]	1	0	1	0	1	1	1	1	-	1	-	7
Dronca [43]	1	0	0	0	1	0	1	1	-	1	-	5
Ferretti [44]	1	1	0	0	1	1	1	1	-	1	-	7
Göçmen [45]	1	0	1	0	1	1	1	1	-	1	-	7
Lahrach [46]	1	0	0	0	1	1	1	1	-	1	-	6
Prakash [47]	1	1	0	0	1	1	1	1	-	1	-	7
Saeed [48]	1	0	0	0	1	1	1	1	-	1	-	6
Senol [49]	1	1	0	0	1	1	1	1	-	1	-	7
Moradi [37]	1	1	0	0	1	1	1	1	-	1	-	7
Paragh [40]	1	0	0	0	1	1	1	1	-	1	-	6
Varga [41]	1	1	0	0	1	1	1	1	-	1	-	7
Abdin [35]	1	1	0	0	1	1	1	1	-	1	-	7
Kannampuzha [36]	1	0	0	0	1	1	1	1	-	1	-	6
Moradi [37]	1	1	0	0	1	1	1	1	-	1	-	7
Rajković [38]	1	0	0	0	1	1	1	1	-	1	-	6
Emre [28]	1	1	0	0	1	1	1	1	-	1	-	7
Gugliucci [29]	1	1	0	0	1	1	1	1	-	1	-	7
Horoz [30]	1	1	1	0	1	1	1	1	-	1	-	8
Johnson-Davis [31]	1	0	0	0	1	1	1	1	-	1	-	6
Kimak [32]	1	1	0	0	1	1	1	1	-	1	-	7
Kuchta [33]	1	1	0	0	1	1	1	1	-	1	-	7
Cacciagiú [20]	1	1	1	0	1	1	1	1	-	1	-	8
Gbandjaba [25]	1	1	0	0	1	1	1	1	-	1	-	7
Mahrooz [26]	1	1	0	0	1	1	1	1	-	1	-	7
Ribeiro [27]	1	1	0	0	1	1	1	1	-	1	-	7
Sztanek [15]	1	1	0	0	1	1	1	1	-	1	-	7
Kolarz [21]	1	1	0	0	1	1	1	1	-	1	-	7
Locsey [22]	0	0	0	0	1	0	1	0	-	1	-	3
Gugliucci [20]	1	1	0	0	1	1	1	1	-	1	-	7
Abdallah [19]	1	1	0	0	1	1	1	1	-	1	-	7
Coimbra [16]	1	1	0	0	1	1	1	1	-	1	-	7
Suematsu [18]	1	1	1	0	1	1	1	1	-	1	-	8
Sridevi [17]	1	0	0	0	1	1	1	1	-	1	-	6
Jose [13]	1	1	1	0	1	1	1	1	-	1	-	8
Szentimrei [15]	1	1	0	0	1	1	1	1	-	1	-	7
